# Phylogenetically informed logic relationships improve detection of biological network organization

**DOI:** 10.1186/1471-2105-12-476

**Published:** 2011-12-15

**Authors:** Jike Cui, Todd F DeLuca, Jae-Yoon Jung, Dennis P Wall

**Affiliations:** 1 Center for Biomedical Informatics, Harvard Medical School, Boston, MA 02115 USA

## Abstract

**Background:**

A "phylogenetic profile" refers to the presence or absence of a gene across a set of organisms, and it has been proven valuable for understanding gene functional relationships and network organization. Despite this success, few studies have attempted to search beyond just pairwise relationships among genes. Here we search for logic relationships involving three genes, and explore its potential application in gene network analyses.

**Results:**

Taking advantage of a phylogenetic matrix constructed from the large orthologs database Roundup, we invented a method to create balanced profiles for individual triplets of genes that guarantee equal weight on the different phylogenetic scenarios of coevolution between genes. When we applied this idea to LAPP, the method to search for logic triplets of genes, the balanced profiles resulted in significant performance improvement and the discovery of hundreds of thousands more putative triplets than unadjusted profiles. We found that logic triplets detected biological network organization and identified key proteins and their functions, ranging from neighbouring proteins in local pathways, to well separated proteins in the whole pathway, and to the interactions among different pathways at the system level. Finally, our case study suggested that the directionality in a logic relationship and the profile of a triplet could disclose the connectivity between the triplet and surrounding networks.

**Conclusion:**

Balanced profiles are superior to the raw profiles employed by traditional methods of phylogenetic profiling in searching for high order gene sets. Gene triplets can provide valuable information in detection of biological network organization and identification of key genes at different levels of cellular interaction.

## Background

Phylogenetic relationships among gene sequences have been used to reconstruct the evolution and organization of gene functional networks in cells. Profiles of orthologous relationships among genomes, specifically the presence or absence of genes across a set of organisms, have proved to be valuable for understanding functional relationships and network organization [1].

The practice of "phylogentic profiling" has successfully penetrated many efforts in comparative genomics including genome annotation and functional coevolution [2-6]. The accuracy and predictive power tends to be modest unless the profiles are reconstructed in a way that accounts for phylogenetic relationships among the genomes, as demonstrated by a few examples [7-10]. Most likely due to the computational complexity, few studies have attempted to search beyond just pairwise relationships among proteins. Yet, given the nature of cellular complexity and adaptive coevolution, we should expect that high order relations exist that are reflected in the pattern of presence and absence of multiple proteins. Indeed, Bowers et al. invented Logic Analysis of Phylogenetic Profiles (LAPP) and demonstrated the clear benefits of identifying relationships among gene triplets, as these have greater likelihood of yielding the network organization and providing more information (in particular directionality) about the nature of the interactions among triplets of genes [11-13]. Recently the concept of gene triplets was applied to gene expression data to study the coordinated regulation of multi-protein complex [13].

With hundreds of genomes spanning a large quadrant of the tree of life, we are now uniquely poised to revisit this hypothesis to determine if higher order logic relationships exist among phylogenetic profiles that are reflective of underlying biological networks and that yield actionable predictions for improved understanding of cellular organization. An important consideration that should have great impact on our ability to decipher complex relationships among triplets of genes is that the network structures and patterns of adaptive coevolution vary across the phylogeny, and thus how the phylogeny is sampled and how it is integrated into the profile comparison to make predictions dramatically impact the accuracy [2,7-10]. Here we infuse phylogeny directly into the search for higher order logical relationships, specifically gene triplets. Based on the phylogenetic matrix constructed from a large orthologs database Roundup [14,15], we balanced phylogenetic profiles for each gene triplets so that each scenario of coevolution between genes is equally weighted when computing their logic relationships. After applying our method into LAPP, we observed significant performance improvement over profiles with no adjustment on their phylogenetic compositions. We then studied the potential benefit of using logic triplets to detect the organization of biological network, and concluded that it could be a very useful tool in deriving the structure, connectivity, and key proteins in cellular networks.

## Results

### Phylogenetic balance among profiles yields greater detection of biological networks

We selected 182 bacterial genomes from the orthologous gene database Roundup [14] and generated a matrix of phylogenetic profiles containing 13,673 rows (see Additional file 1). Each row represented a gene and contained a binary string of 0's and 1's to indicate the presence or absence of that gene in the set of 182 genomes. We then randomly selected millions of triplets of genes. Through empirical investigation of these triplets, we learned that the genome composition had a tendency to introduce biases towards specific coevolutionary outcomes, especially for the common and rare genes. In any given organism, there are four such possible outcomes, as listed in Table 1, between the genes *a *and *b *within a triplet containing genes *a, b*, and *c*.

**Table 1:** Four evolutionary scenarios between the genes a and b within a triplet containing genes a, b, and c

	Scenario 1	Scenario 2	Scenario 3	Scenario 4
Gene *a*	0	0	1	1

Gene *b*	0	1	0	1

If any one scenario is heavily overrepresented because of the genomic composition of the phylogenetic profiles used to decipher logic relationships, and ultimately, to predict network structure, our ability to detect certain logic relationships over others was concomitantly compromised. Through our inspection of the compendium of triplets generated from the 182 genomes, we learned that this was indeed the case. In each *ab *pair, the average number of genomes containing one of the four possible evolutionary scenarios was 45, with an average standard deviation of 44.8. We reasoned that such imbalance among the four scenarios would lead to increased rates of both false positives and false negatives in our predictions.

To address this directly, we devised a strategy to create balanced profiles for each triplet of genes such that the four scenarios of coevolution were equally weighed in those specific profiles, as detailed in the Methods. We then tested whether these balanced phylogenetic profiles yielded greater accuracy than our original profiles containing varying weights on the four scenarios of coevolution between genes. To do so, we computed *ΔU*, a measure of deviation in entropy that ranges from -1 to 1 and, when high, indicates the presence of a nonrandom functional relationship among the three genes in question (See Methods) [11,13], and mapped the predicted triplet relationships to known Biological Process annotations from Gene Ontology (GO). We considered a high scoring triplet with *ΔU ≥ 0.3 *to be statistically significant significance (see the "Statistic significance of *ΔU*" in the Additional file 2 for detail), and called a triplet putative when its three constituent genes contained the same annotation from GO.

**Figure 1:**
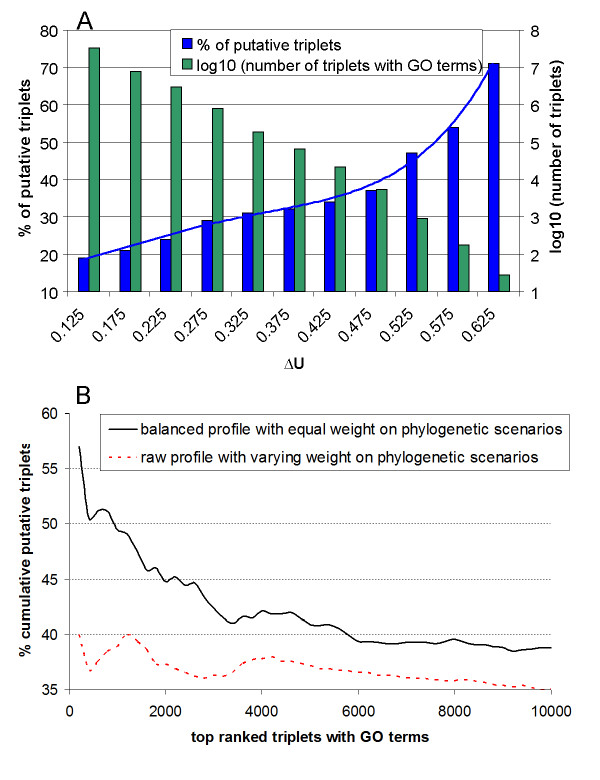
**Balanced profiles outperforms the raw profiles**. Compared to raw profiles with varying weights on the four phylogenetic scenarios of coevolution, balanced profiles with equal weight on those scenarios improve the prediction accuracy and identify many-fold more putative triplets as well. *Triplets with GO terms *refers to triplets where GO terms are available for all three genes, and putative triplets are those triplets where the three genes share common GO terms. The percentage of putative triplets in all triplets with GO terms is a measure of prediction accuracy. **A**: *ΔU *is a score to signal the significance of a triplet, ranging from -1 (very weak) to 1 (very strong). A bar at *ΔU *of 0.125 refers to the region [0.10, 0.15). The number of predicted triplets decreases exponentially with the increase of *ΔU*. And there is a strong positive correlation (R^2 ^= 0.99) between the *% of putative triplets *and *ΔU*, an evidence that *ΔU *indeed signals functional relatedness of genes. B: Ranked in the descending order of *ΔU*, the top triplets computed from balanced profiles consistently display a higher tendency to have functional relations within their three genes than the raw profile, indicating the benefit of giving equal weight to the four scenarios of coevolution between genes. In addition, substantially more triplets are produced from balanced profiles than the raw profiles without sacrifice on accuracy.

Our computation resulted in 284,498 triplets with GO terms available for three genes and *ΔU ≥ *0.3. With the increase of *ΔU*, the number of predicted triplets decreases exponentially (Figure 1A). We used the percentage of putative triplets in all triplets where GO terms are available for all three genes as a measure of accuracy in the prediction of triplets. Figure 1A shows that there is a strong positive correlation between the percentage of putative triplets and *ΔU*, suggesting that *ΔU *indeed infers relatedness in biological functions in addition to its statistical significance.

To compare the performance between the balanced profile with equal weight on phylogenetic scenarios of coevolution and the raw profile with varying weights on those scenarios, we ranked the triplets in the descending order of *ΔU *and compared the top 10,000 triplets predicted using the two types of profiles, which is a common way of evaluating different methods in the prediction of gene pairwise relationships [2]. Figure 1B shows that the balanced profiles consistently lead to higher accuracy (the % accumulative putative triplets) in the assignment of the triplets than the raw profiles. At the same level of accuracy, the balanced profiles produce many more triplets as well.

**Figure 2:**
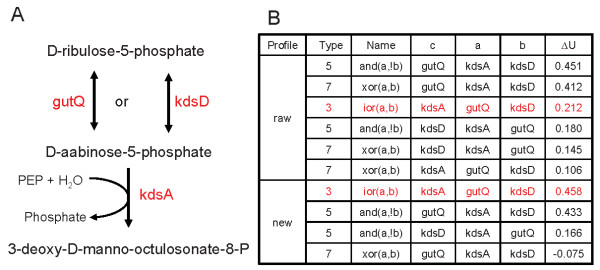
**An example illustrating the benefit of balanced profiles**. Balanced profiles guarantee equal weight on the four scenarios of coevolution between genes, while raw profiles contain varying weights. **A**: The first two steps in CMP-KDO biosynthesis I in *E. coli *[20]. Enzymes gutQ and kdsD are both D-arabinose-5-phosphate isomerase; enzyme kdsA is 3-deoxy-D-manno-octulosonate-8-phosphate synthase, and PEP is phosphoenolpyruvate. The first step can be catalyzed by either gutQ or kdsD, and the second step by kdsA. The three genes form a type 3 logic relationship, "*kdsA *is present in a genome if and only if *gutQ *or *kdsD *is present". **B**: Using raw profiles, two significant triplets are identified with *ΔU *> 0.3, along with four other triplets with ΔU > 0.1. Neither of the two significant triplets is consistent with the network in *E. coli *or has experimental support, and the expected type 3 triplet only ranks third with a low insignificant *ΔU*. In comparison, the balanced profiles produce top score for the expected triplet, significantly lower the score of an unfitting triplet from 0.412 to -0.075, and identify only three triplets with ΔU > 0.1; that is, much less noise.

Figure 2 provides further intuition as to why the balanced profiles in which the evolutionary scenarios shown above were given equal weight enabled such a large increase in prediction accuracy. Under this example, the balancing of the phylogenetic profiles enabled detection of the biologically proven triplet relationship "*kdsA *is present in a genome if and only if (iff) *gutQ *or *kdsD *is present". In contrast, the unadjusted phylogenetic profiles with varying weight among the four phylogenetic scenarios of coevolution detected a logic relationship, "*gutQ *is present iff *kdsA *is present and *kdsD *is not", that is inconsistent with the known network in *E. coli*. Though that relationship might be present in some bacteria, the outcome was high confidence in a prediction that is contrary to known networks and has no experimental support so far. Balancing of the phylogentic profiles also filtered out most of the other unlikely triplets predicted with the raw profiles, notably lowering the *ΔU *from 0.412 to -0.075 for relationship type 7 "*gutQ *is present iff one of *kdsA *and *kdsD *is present", a completely unfitting description for Figure 2A.

As Additional file 2, Figure S1 shows, the top three most frequent logic relationships across the whole spectrum of *ΔU *are type 5, 1, and 3 in Table 2; type 7 is rare, which might be partially due to the exclusion of eukaryotes and archaea in our profiles.

**Table 2:** Eight logic relationships among three genes *a, b*, and *c *[11]

Type	Description	logic symbols
1	*c *is present in a genome iff *a *and *b *are both present.	*c *↔ *a *∧ *b*

2	*c *is present in a genome iff *a *is absent or *b *is absent.	*c *↔ ^~^(*a *∧ *b*)

3	*c *is present in a genome iff *a *is present or *b *is present.	*c *↔ *a *∨ *b*

4	*c *is present in a genome iff *a *is absent and *b *is absent.	*c *↔ ^~^(*a *∨ *b*)

5	*c *is present in a genome iff *a *is present and *b *is absent.	*c *↔ *a *∧^~^*b*

6	*c *is present in a genome iff *a *is absent or *b *is present.	*c *↔ ^~^*a *∨ *b*

7	*c *is present in a genome iff one of either *a *or *b *is present.	*c *↔ *a *⊕ *b*

8	*c *is present in a genome iff *a *and *b *are both present or both absent.	*c *↔ ^~^(*a *⊕ *b*)

Among other possible logic relationships involving three genes, the above eight are most likely to be present in real cellular networks. *iff *stands for *if and only if*.

### Gene triplets reveal biological network at different levels and key proteins at each level of interaction

Any cellular network is the result of millions years of evolution, and the power of logic triplets is that it can follow the path of the evolution and deduce the present information on the local, middle, and system levels of the network.

**Figure 3:**
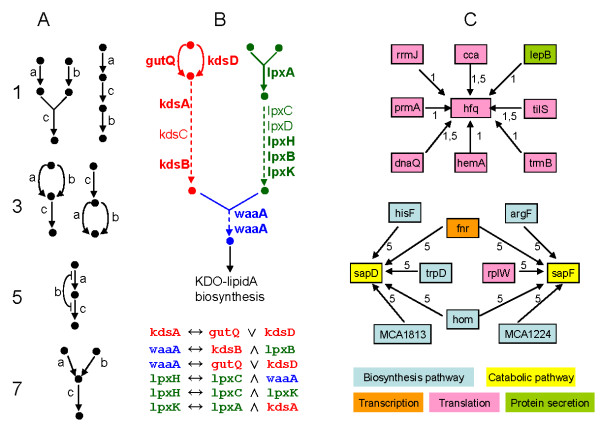
**Gene triplets reveal biological networks at different levels and key genes in those networks**. A: The basic network structures for logic types 1 (*c *↔ *a *∧ *b*), 3 (*c *↔ *a *∨ *b*), 5 (*c *↔ *a *∧^~^*b*), and 7 (*c *↔ *a *⊕ *b*); black dots represent chemical compounds. Type 5 means protein *b *may inhibit enzyme *a *or *c*. These structures can be applied locally to infer protein function, or on a larger scale to reveal protein network organization. **B**: An example illustrating the power of logic triplets to deduce the network of KDO_2_-lipidA synthesis [20]; dashed line represents a series of sequential steps catalyzed by different enzymes on different intermediate compounds. The pathway consists of three parts: i) CMP-KDO biosynthesis I in red; ii) lipid IV_A _biosynthesis in green; and iii) KDO transfer to lipid IV_A _I in blue. The listed logic triplets convincingly disclose the structure of the whole pathway, the order of enzymes, and even key proteins (in bold font). **C**: Logic triplets also reveal the interaction among different pathways in the whole cellular network. The upper diagram indicates that RNA-binding protein hfq regulates gene expression and protein secretion. The lower diagram illustrates the interaction among the major pathways in a cell. Numbers denote the type of logic relationships, and arrows point to the *c *genes in logic triplets.

Figure 3A illustrates the major network structure compatible to the four main logic relationships 1, 3, 5, and 7 (See Table 2). The structure 1 and 7 are same as those developed by Bowers et al. [11], but 3 and 5 are different and new. The type 5 structure suggests that enzyme *b *inhibits the step from enzyme *c *to *a*, which can be achieved through multiple mechanisms, such as enzyme *b*'s products reducing the activities of enzymes *c *or *a, b *binding with the mRNAs of *c *or *a *to lower their expression, *b *competing with *c *on the substrates, etc. Applied inside each pathway, the relationships can tell the local structure of the pathway and the functions of local proteins. Taking triplets in Additional file 2, Figure S2 as examples, the triplet "*cobS *is present iff *cobU *and *cobC *are both present" declares that cobS requires two substrates produced separately by cobU and cobC; the triplet "*hisB *is present iff either *hisF *or *hisH *is present" suggests that hisF and hisH may have similar functions and both can produce the substrate for hisB; however, type 3 relationship can also indicate that enzyme *c *produces a substrate for enzymes *a *and *b*, as in the case "*hemE *is present iff either *hemF *or *hemN *is present". Besides those basic structures, there may be other compatible but uncommon structures fitting those logic types.

Logic triplets can catch not only the relationship among neighboring proteins, but also those well separated in a local pathway. Figure 3B gives an excellent example. 12 genes are involved in that part of the KDO_2_-lipidA synthesis pathway, constituting 74 significant triplets within each of the green and red sections and among the three sections (as compared to only seven significant triplets found without balancing the profiles). The whole pathway can almost be deduced accurately from the large number of triplets. For example, triplets "*kdsA/B *is present iff either *gutQ *or *kdsD *is present" tell the structure of the red branch; and triplets like "*lpxH *is present iff both *lpxC *and *lpxK *are present" signal the clustering of those genes in the green branch; triplet "*waaA *is present iff both *kdsB *and *lpxB *are present" indicates the mergence of the two branches, which is further strengthened by triplets "*lpxH *iff *lpxC *and *waaA*" and "*waaA *iff *gutQ *or *kdsD*".

On an even higher level, the interaction among different pathways in the whole cellular network can also be disclosed using logic triplets, some of which may be very difficult to examine otherwise. Figure 3C suggests that RNA-binding protein hfq interacts with many other proteins in translation, and surprisingly with lepB, a signal peptidase as well. However, those relationships agree with other studies which suggest hfq stimulates sRNA-mRNA pairing and regulates gene expression and protein secretion [16-18]. Furthermore, Figure 3C points out the key proteins involved in the coupling between the regulation of translation and protein secretion. Another example is the interaction among the catabolic pathway, the biosynthetic pathway, transcription, and translation; they are expected to coordinate, but logic triplets shed light on how they function together and what the key proteins for such coordination are.

We also studied the relationship among the number of triplets a gene was involved in, its role in the triplets (*c *or *a, b*), and its essentiality in *E. coli*. We found that the essentiality of a gene was significantly related to its likelihood to be in significant triplets and was associated with its roles in triplets. Essential genes were more likely to be in significant triplets and be in the input role (*a *or *b*), whereas non-essential genes were more likely to be in the output role (position *c*). Please see the section "Triplets and Gene Essentiality" in Additional file 2 for details. In addition, we investigated genes and GO terms with most triplets, as listed in the section "Enriched Genes and GO Terms" in Additional file 2.

## Discussion

### The underlying conditions critical for a logic triplet

**Figure 4:**
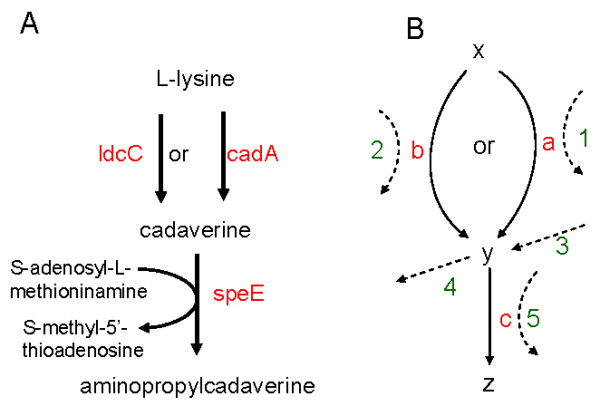
**Illustrations for the underlying conditions of a logic triplet**. A: Aminopropylcadaverine biosynthesis in *E. coli *[20]. Enzymes ldcC and cadA are lysine decarboxylases, and speE is an aminopropylcadaverine synthase. The first step can be catalyzed by either ldcC or cadA depending on the reaction conditions. However, the three genes only form an *if *instead of a full *iff *logic triplet because of the presence of path 4 and 5 in Figure 4B. **B**: The underlying conditions for the logic triplet "*c *is present iff *a *or *b *is present". Compound *x *can be converted to compound *y *by either enzyme *a *or *b, y *can then be catalyzed by enzyme *c *to produce compound *z*. Dashed lines represent other reactions marked by numbers; 1, 2, and 5 mean the enzymes can catalyze other reactions, 3 and 4 mean *y *can be the product or substrate in other paths. The underlying conditions for the *iff *logic triplet are that none of the five dashed lines can be present in large number of species; that is, *a, b, c*, and *y *cannot often be involved in other type of reactions.

Although the implementation of balanced phylogeny of coevolution into the new profiles helped to recover many more triplets, only a third of *E. coli *genes were found to have significant triplets with *ΔU *≥ 0.3; the remaining two-thirds had smaller or negative *ΔU*. A low *ΔU *value is caused by the genomes with *abc *coevolutionary scenario incompatible with the logic relationships. For the example in Figure 4A, *abc *coevolutionary scenarios of 001, 100, and 010 violate the logic relationship "*speE *is present iff *ldcD *or *cadA *is present". When large number of genomes have such violation, as the case in Figure 4A, the *ΔU *will be very small. While the reasons for the presence of incompatible genomes could be horizontal gene transfer [19] and errors in genome sequencing, compilation, ortholog identification and clustering algorithms, an important factor could be the directionality of the relationship which reflects the degree of interaction between the members of triplets and surrounding network.

In Figure 4A, compatible *abc *coevolutionary scenarios are 000, 011, 101, and 111. As explained in the "The bi-directionality of the *iff *condition in a logic triplet" in the Additional file 2, the *iff *condition in a logic triplet is bi-directional, meaning that *ab *determines *c*, and *c *determines *ab *as well. For instance, the two directions in the *abc *coevolutionary scenario 000 reflect the interaction with surrounding network as illustrated in Figure 4B. In the forward direction, if genes *a *and *b *are absent, gene *c *is absent too; it says that path 3 and 5 do not exist, meaning that compound *y *is not synthesized from any other paths and that enzyme *c *is not involved with other reactions either. That is because enzyme *c *can still function without *a *or *b *if path 3 or 5 is available and thus gene *c *will be under evolutionary selection and be kept during the course of evolution. Conversely in the reverse direction, if gene *c *is absent, genes *a *and *b *are absent as well; the implication is that path 1, 2, and 4 do not exist, which suggests that none of *a, b*, and *y *is in other types of reactions for the similar reason in gene evolution. In brief, the bi-directionality of the logic relationship sets the condition of closure that the enzymes and middle compounds involved in the association appear only in that type of local path. If the condition of closure is not met, the logic relationship may change from *iff *to *if*, and genomes with *abc *coevolutionary scenarios incompatible with the full *iff *logic relationship become common, leading to a small *ΔU*. That could be why the difference between the percentage of putative triplets is small within 0.25 ≤ *ΔU *≤ 0.45 in Figure 1A; that is because many triplets with small *ΔU *are likely true partial triplets with *if *instead of *iff *relationship.

In the triplet of Figure 2A, all the five paths in Figure 4B are likely to be absent [20], satisfying the condition of closure; as a result, the triplet had very few genomes with incompatible *abc *coevolutionary scenarios, leading to a high *ΔU*. Conversely, path 4 and 5 are present in the triplet of Figure 4A, causing incompatible *abc *coevolutionary scenarios of 001, 010, and 100, and consequently low *ΔU *for the triplet. Figure 4 gives hints to develop a method to identify a partial logic relationship, such as the *if *logic relationship, which is likely quite common in a real cellular system. With phylogenetically representative genomes, the *ΔU *and the number of genomes with different *abc *coevolutionary scenarios could infer the status of the five paths in Figure 4B. A high *ΔU *indicates the absence of the five paths, and a low *ΔU *suggests the presence of some paths. In Figure 4A, the only incompatible *abc *coevolutionary scenario with large number of genomes is 001, which infers the presence of path 3 or 4 or 5. And indeed, path 4 and 5 exist [20]. For example, speE also catalyzes the biosynthesis of spermidine from putrescine and S-adenosyl-L-methioninamine. That type of information can be very valuable in the construction of metabolic network.

Due to the dramatic difference between eukaryotes and prokaryotes in the complexity and connectivity of cellular networks, probably only a small number of gene triplets can maintain such a condition of closure consistently across both domains; therefore, we did not mix them in our study.

### Logic relationships can span across different taxonomic phyla or domains

In the study of gene pairwise relationships using phylogenetic profiling, the focuses are the co-occurrence and co-absence between two genes. That restricts the relationships to be within individual genomes. Since our method gives equal weight to all of the four scenarios of coevolution, it has the power to detect relationships across different phyla of organisms. In other words, all the three genes in a logic triplet do not have to be present in the same species. A good example is the discovery of *VKOR*, a functional ortholog of gene *dsbB *that has the same function but shares no sequence similarity with *dsbB*, by Dutton et al [21]. Enzyme dsbB functions with enzyme dsbA to form disulfide bonds in most bacteria but is absent in several major bacterial phyla containing *dsbA *and disulfide bonds. Dutton et al. selected *VKOR *as a candidate for the role of *dsbB *through experiments and finally proved that by unknowingly constructing a type 3 logic relationship "*dsbA *is present iff either *dsbB *or *VKOR *is present"

## Conclusions

In this study, we used phylogenetic matrix of 182 bacterial genomes to search gene triplets with eight types of logic relationships. To measure how well a logic relationship holds among all phylogenetic outcomes, we created a balanced profile with equal weight on the four scenarios of coevolution for each triplet. The new profiles significantly improved the accuracy of prediction over raw profiles, and discovered many more putative triplets.

Logic triplets have great power in detecting the structure of biological network at different levels, identifying key proteins at each level of interaction, and give inference to the function of proteins. In some cases, parts of a pathway can be deduced in high resolution. In addition, they can also infer the status of other paths where the proteins and compounds in a triplet are involved. Those can be very helpful in the derivation of gene and protein networks with details; future work will follow that direction.

Searching for higher order relationships would require extremely intensive computation, but with a good algorithm and a limited set of genes such computations would be possible and of great value to our understanding of cellular networks. Such relationships, combined with binary and ternary functional gene sets, might be able to derive gene networks with high accuracy. Finally, the method can help to identify gene functional orthologs bearing no sequence similarity.

## Methods

### Selecting representative genomes and constructing raw profiles

We selected 182 genomes from Roundup version 2010_01 [14,15] (Additional file 2, Table S1). Representatives were chosen based on their taxonomic classification in NCBI; for multiple genomes in the same family, the one with most genes was selected. Then using the reciprocal smallest distance algorithm implemented in Roundup to identify orthologs [15], a phylogenetic matrix consisting of 0s and 1s was constructed with *Escherichia coli K 12 substr MG1655 *as a reference genome, where the rows were genes and columns were species; 0 indicated absence of a gene in a genome, and 1 indicated presence. Hamming distance was computed between any two columns. If the distance was smaller than 5% of the total number of rows, the species with more zeros was removed so that the representatives were well spread phylogenetically. Then the matrix was recomputed for the remaining species without any reference genome, and rows with identical profiles were clustered.

### Finding logic relationships

Multiple metrics, such as Pearson's correlation, hypergeometric p-values, and mutual information [6,22,23], have been proven effective in measuring the correlation between two phylogenetic profiles. However, none of them can infer the directionality in the network structure among three genes. By comparison, Uncertainty Coefficient, invented by Bowers et al., introduces directionality into dependency computation between two genes [11]. A more detailed mathematic overview of the metric is given in the Supplemental Methods of a recent paper by the group [13]. Here is a short description of U, the Uncertainty Coefficient, for readers' convenience.

$$\begin{array}{ccc} U\left(a|b\right) & =I\left(a,b\right)∕H\left(a\right)\;\; & \\ & =\;\;\left[H\left(a\right)+H\left(b\right)-H\left(a,b\right)\right]∕H\left(a\right) \end{array}$$

Where *a, b*, and *c *represent the profiles of gene *a, b*, and *c *in the phylogenetic matrix. *U(a|b) *is the Uncertainty Coefficient that measures the functional dependency of gene *a *on gene *b*. *I(a, b) *is mutual information of two profiles, *H(a) *is the entropy of profile *a*, and *H(a, b) *is the joint entropy of two profiles.

The value of *U *lies between 0 and 1, where 0 indicates no dependency and 1 indicates complete dependency of *a *on *b*. (In this sense, Certainty Coefficient should be a more appropriate name for U). *ΔU *= *U(c|f(a, b) - max(U(c|a), U(c|b)) *is used to represent the significance of the logic relationship *f*; it is simply the difference between the dependency of *c *on the logic function *f *(*a, b) *and its dependency on *a or b *individually.

In our study, we used *ΔU *to search the eight triplet relationships (Table 2) which were developed and suggested to have high frequency in real biological networks by Bowers et al [11]. Statistically, higher *ΔU *corresponds to smaller p-value and higher confidence on the triplet prediction (see the "Statistic significance of *ΔU*" in the Additional file 2 for detail). In addition, the Results section shows that there is a strong positive correlation between *ΔU *and the functional relatedness among the three genes in a triplet.

The next four paragraphs describe our invention of balanced phylogenetic profile, which, as the Results section suggests, improves both the quality and quantity of predicted logic triplets when applied to Bowers's method.

Using notations same as those in a phylogenetic profile, let 0 represent the absence of a gene in a genome, and 1 represents presence. There are four possible phylogenetic scenarios of coevolution (00, 01, 10, 11) for two genes *a *and *b*, and similarly eight scenarios (000/1, 010/1, 100/1, 110/1) for three genes *a, b*, and *c*. Among the 182 genomes in the construction of phylogenetic matrix from Roundup, for *i, j, k *∈ {0, 1}, let *p_ij _*be the proportion of genomes containing the four possible scenarios of coevolution, and let *p_ijk _*be the proportion of genomes matching each of the eight possible scenarios.

*U(c|f(a, b)) *evaluates the relationship between *c *and the logic function of the four scenarios. As noted in the Supplemental Methods of the recent study [13], the uncertainty coefficient is a function of *p_ijk_*. To well-determine a Boolean function f(a, b), one needs to know f(0,0), f(0,1), f(1,0), and f(1,1); hence, to judge c = f(a, b) one needs to consider the observed proportion of c = 0 vs. c = 1 for each of observed ab = 00/01/10/11. However, for any given triple of genes, the partition of organisms according to observed ab = 00/01/10/11 may have more of one of the four outcomes than others. This variation in *p_ij _*equals to giving different weights to each of the four coevolutionary scenarios between genes *a *and *b*, which leads to erroneous *ΔU*.

For example, consider the type 3 logic relationship "gene *c *is present iff one of either genes *a *or *b *is present." For it to hold, ideally *p_000 _*> >*p_001_, p_011 _*> >*p_010_, p_101 _*> >*p_100_*, and *p_110 _*> >*p_111_*. However, a triplet with *p_000 _*< <*p_001_*, which is against type 3 relationship, can still have a high *ΔU *for such a relationship if *p*_*00 *_< < 0.25. Our data in the Results section suggest that it is critical to weigh the four scenarios equally in the computation of *U(c|f(a, b)) *in order to correctly identify the logic relationships.

Therefore, in order to guarantee that all the phylogenetic scenarios are equally weighted, we computed balanced profiles with *p'_ijk _*for each triplet so that *p'_ij0_*/*p'_ij1 _*= *p_ij0_*/*p_ij1 _*and all *p'_ij _*are equal. This ensures that all the four scenarios of coevolution between gene *a *and *b *are weighed equally in the computation of *ΔU*. We also skipped profiles with fewer than 18 genomes for its 0 or 1 and triplets which had fewer than 18 genomes for any of the four scenarios, because they contained too little entropy and might not be informative.

**Figure 5:**
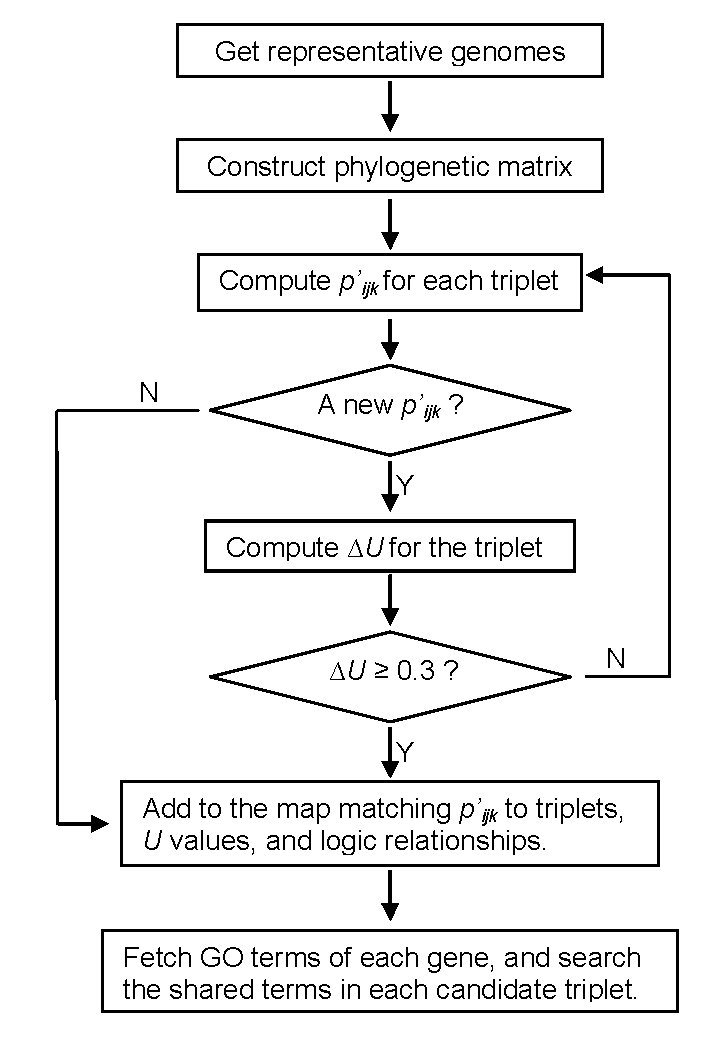
**Computational flow in the search of logic triplets of genes**. 182 representative bacteria genomes were selected from the 770 bacteria genomes available in Roundup [14,15], and orthologs were identified using the RSD (Reciprocal Smallest Distance) algorithm [15]. The final matrix contained 13,673 gene profiles. In the computation of the logic triplets, *p'_ijk_*, the balanced profile was created to give equal weight on the four scenarios of coevolution between genes *a *and *b*. *ΔU *was taken as the significance of a triplet; a value greater than 0.3 was deemed significant. The percentage of triplets where the three genes share common GO terms was used to measure the accuracy of the prediction.

Figure 5 outlines the primary components of our algorithm to identify logic relationships among the profiles.

### Performance Evaluation

Sharing pathways or GO terms has been used to measure the functional relatedness between genes [10]. Here we apply the same principle and consider a triplet putative if its three genes have common GO terms in Biological Process. Thereby we employed the following definition:

The accuracy of triplet assignment = N_putative_/N_all_, where N_putative _is the number of putative triplets, and N_all _is the number of triplets where the three members all have GO terms available. Those triplets where one or more members did not have GO terms were excluded.

As the Results and Discussion sections show, genes in such logic triplets may locate in different pathways, and even in different genomes or phyla. In addition, genes in the same pathway may not all have GO terms available at the same level. Because of that, the ancestors of each GO term up to four levels under the root were also included, which is the level for a set of similar pathways. The ancestor of a GO term was identified by GO graph_path table. We conducted no further filtering by link type or evidence code, no special treatment of synonyms and/or obsolete GO terms.

GO was acquired by downloading the go_*-assocdb-data.gz file from ftp://ftp.geneontology.org/pub/go/godatabase/archive/latest-full/ around Sept. 2010. GI IDs were mapped to GO terms by gene2go table downloaded from ftp://ftp.ncbi.nlm.nih.gov/gene/DATA/gene2go.gz around Sept. 2010.

## Authors' contributions

DW and JC designed the study and wrote the manuscript. JC, TD, and JJ carried out the study. TD helped to proof read the draft. All authors read and approved the final manuscript.

## Supplementary Material

Additional file 1 **The phylogenetic profile matrix used in our study**. Click here for file

Additional file 2 **It is a PDF file and includes the following content: 1. Supplemental table 1**. The taxonomic distribution of the 182 bacteria genomes. 2. Supplemental table 2. Top ten genes involved in most triplets and their functions. 3. Supplemental figure 1. Percentage of different logic relationships among the eight types across the whole spectrum of ΔU. 4. Supplemental figure 2. Some examples of logic triplets in E. coli. 5. Supplemental figure 3. The log number of triplets where a gene is in position *c *vs. *a *or *b*. 6. Results and discussion on Gene triplets and gene essentiality. 7. Results and discussion on Enriched genes and GO terms. 8. Results and discussion on The bi-directionality of the *iff *condition in a logic triplet. 9. Results and discussion on Statistic significance of *ΔU*. Click here for file
