# Supplementary material for: Phylogenetically informed logic relationships improve detection of biological network organization
Source: BMC Bioinformatics. 2011 Dec 15;12:476. doi: 10.1186/1471-2105-12-476 (PMC3402364; doi:10.1186/1471-2105-12-476)
Supplement: Additional file 2 — It is a PDF file and includes the following content: 1. Supplemental table 1. The taxonomic distribution of the 182 bacteria genomes. 2. Supplemental table 2. Top ten genes involved in most triplets and their functions. 3. Supplemental figure 1. Percentage of different logic relationships among the eight types across the whole spectrum of ΔU. 4. Supplemental figure 2. Some examples of logic triplets in E. coli. 5. Supplemental figure 3. The log number of triplets where a gene is in position c vs. a or b. 6. Results and discussion on Gene triplets and gene essentiality. 7. Results and discussion on Enriched genes and GO terms. 8. Results and discussion on The bi-directionality of the iff condition in a logic triplet. 9. Results and discussion on Statistic significance of ΔU. [file 1471-2105-12-476-S2.DOC]

Supplemental table 1. The taxonomic distribution of the 182 bacteria genomes.

| **class name** | **count** | **class name** | **count** |
| --- | --- | --- | --- |
| **Actinobacteria** | **9** | **Elusimicrobia** | **1** |
| **Alphaproteobacteria** | **30** | **Epsilonproteobacteria** | **5** |
| **Aquificae** | **3** | **Fusobacteria** | **1** |
| **Bacilli** | **16** | **Gammaproteobacteria** | **42** |
| **Bacteroidetes** | **4** | **Gloeobacteria** | **1** |
| **Betaproteobacteria** | **18** | **Mollicutes** | **2** |
| **Chlamydiae** | **2** | **Nitrospira** | **1** |
| **Chlorobi** | **1** | **Nostocales** | **1** |
| **Chloroflexi** | **1** | **Planctomycetacia** | **1** |
| **Chroococcales** | **4** | **Prochlorales** | **1** |
| **Clostridia** | **14** | **Spirochaetes** | **3** |
| **Dehalococcoidetes** | **1** | **Thermotogae** | **4** |
| **Deinococci** | **2** | **unified Cyanobacteria** | **1** |
| **Deltaproteobacteria** | **8** | **unified Proteobacteria** | **1** |
| **Dictyoglomia** | **1** | **Verrucomicrobia** | **3** |

The full list:

Escherichia coli K 12 substr MG1655

Acaryochloris marina MBIC11017

Acholeplasma laidlawii PG 8A

Acidiphilium cryptum JF-5

Acidithiobacillus ferrooxidans ATCC 23270

Acidovorax avenae citrulli AAC00-1

Acinetobacter baumannii AB0057

Actinobacillus pleuropneumoniae serovar 7 AP76

Aeromonas salmonicida A449

Agrobacterium radiobacter K84

Akkermansia muciniphila ATCC BAA 835

Alcanivorax borkumensis SK2

Aliivibrio salmonicida LFI1238

Alkalilimnicola ehrlichei MLHE-1

Alkaliphilus metalliredigens QYMF

Alteromonas macleodii Deep ecotype

Anaerocellum thermophilum DSM 6725

Anaplasma phagocytophilum HZ

Anoxybacillus flavithermus WK1

Aquifex aeolicus

Arcobacter butzleri RM4018

Aromatoleum aromaticum EbN1

Arthrobacter chlorophenolicus A6

Azoarcus BH72

Azorhizobium caulinodans ORS 571

Bacillus cereus AH187

Bacteroides thetaiotaomicron VPI-5482

Bartonella tribocorum CIP 105476

Bdellovibrio bacteriovorus

Beijerinckia indica ATCC 9039

Bifidobacterium longum infantis ATCC 15697

Bordetella bronchiseptica

Borrelia burgdorferi

Bradyrhizobium japonicum

Brucella suis 1330

Burkholderia xenovorans LB400

Caldicellulosiruptor saccharolyticus DSM 8903

Candidatus Desulforudis audaxviator MP104C

Carboxydothermus hydrogenoformans Z-2901

Caulobacter K31

Chlamydophila pneumoniae TW 183

Chlorobium phaeobacteroides DSM 266

Chloroflexus aurantiacus J 10 fl

Chromobacterium violaceum

Citrobacter koseri ATCC BAA-895

Clostridium kluyveri DSM 555

Colwellia psychrerythraea 34H

Coprothermobacter proteolyticus DSM 5265

Coxiella burnetii Dugway 7E9-12

Cupriavidus taiwanensis

Cyanothece PCC 8801

Dehalococcoides ethenogenes 195

Deinococcus radiodurans

Desulfatibacillum alkenivorans AK 01

Desulfitobacterium hafniense DCB 2

Desulfotalea psychrophila LSv54

Desulfovibrio desulfuricans G20

Diaphorobacter TPSY

Dichelobacter nodosus VCS1703A

Dictyoglomus thermophilum H 6 12

Dinoroseobacter shibae DFL 12

Ehrlichia chaffeensis Arkansas

Elusimicrobium minutum Pei191

Enterobacter sakazakii ATCC BAA-894

Enterococcus faecalis V583

Erwinia carotovora atroseptica SCRI1043

Exiguobacterium sibiricum 255 15

Fervidobacterium nodosum Rt17-B1

Finegoldia magna ATCC 29328

Flavobacterium johnsoniae UW101

Francisella philomiragia ATCC 25017

Frankia EAN1pec

Fusobacterium nucleatum

Geobacillus kaustophilus HTA426

Geobacter FRC 32

Gloeobacter violaceus

Gluconobacter oxydans 621H

Granulobacter bethesdensis CGDNIH1

Haemophilus parasuis SH0165

Hahella chejuensis KCTC 2396

Halorhodospira halophila SL1

Halothermothrix orenii H 168

Herminiimonas arsenicoxydans

Hydrogenobaculum Y04AAS1

Idiomarina loihiensis L2TR

Janthinobacterium Marseille

Kineococcus radiotolerans SRS30216

Lactobacillus plantarum

Lactococcus lactis cremoris SK11

Legionella pneumophila Paris

Leptospira interrogans serovar Copenhageni

Leuconostoc mesenteroides ATCC 8293

Listeria innocua

Lysinibacillus sphaericus C3 41

Macrococcus caseolyticus JCSC5402

Magnetococcus MC-1

Magnetospirillum magneticum AMB-1

Mannheimia succiniciproducens MBEL55E

Maricaulis maris MCS10

Marinomonas MWYL1

Mesorhizobium loti

Methylacidiphilum infernorum V4

Methylibium petroleiphilum PM1

Methylobacterium radiotolerans JCM 2831

Methylococcus capsulatus Bath

Microcystis aeruginosa NIES 843

Moorella thermoacetica ATCC 39073

Mycoplasma penetrans

Myxococcus xanthus DK 1622

Nautilia profundicola AmH

Neisseria gonorrhoeae NCCP11945

Nitratiruptor SB155-2

Nitrobacter hamburgensis X14

Nitrosococcus oceani ATCC 19707

Nitrosomonas eutropha C71

Nostoc punctiforme PCC 73102

Oceanobacillus iheyensis

Oenococcus oeni PSU-1

Oligotropha carboxidovorans OM5

Opitutus terrae PB90 1

Orientia tsutsugamushi Ikeda

Parabacteroides distasonis ATCC 8503

Parachlamydia sp UWE25

Paracoccus denitrificans PD1222

Pasteurella multocida

Pediococcus pentosaceus ATCC 25745

Pelobacter carbinolicus

Petrotoga mobilis SJ95

Phenylobacterium zucineum HLK1

Photobacterium profundum SS9

Photorhabdus luminescens

Pirellula sp

Polaromonas naphthalenivorans CJ2

Polynucleobacter necessarius asymbioticus QLW P1DMWA 1

Prochlorococcus marinus MIT9313

Propionibacterium acnes KPA171202

Pseudoalteromonas atlantica T6c

Pseudomonas aeruginosa PA7

Psychrobacter cryohalolentis K5

Psychromonas ingrahamii 37

Ralstonia eutropha JMP134

Rhodobacter sphaeroides KD131

Rhodococcus jostii RHA1

Rhodoferax ferrireducens T118

Rhodopseudomonas palustris BisB18

Rhodospirillum centenum SW

Saccharophagus degradans 2-40

Saccharopolyspora erythraea NRRL 2338

Salinibacter ruber DSM 13855

Salinispora arenicola CNS-205

Salmonella enterica serovar Paratyphi B SPB7

Shewanella woodyi ATCC 51908

Shigella dysenteriae

Sinorhizobium meliloti

Sodalis glossinidius morsitans

Sphingomonas wittichii RW1

Sphingopyxis alaskensis RB2256

Staphylococcus aureus NCTC 8325

Streptococcus sanguinis SK36

Streptomyces coelicolor

Sulfurihydrogenibium YO3AOP1

Sulfurovum NBC37-1

Symbiobacterium thermophilum IAM14863

Synechococcus PCC 7002

Syntrophomonas wolfei Goettingen

Syntrophus aciditrophicus SB

Thermoanaerobacter tengcongensis

Thermodesulfovibrio yellowstonii DSM 11347

Thermosipho africanus TCF52B

Thermosynechococcus elongatus

Thermotoga lettingae TMO

Thermus thermophilus HB8

Thiobacillus denitrificans ATCC 25259

Thiomicrospira crunogena XCL-2

Treponema denticola ATCC 35405

Vibrio harveyi ATCC BAA-1116

Wolbachia endosymbiont of Culex quinquefasciatus Pel

Wolinella succinogenes

Xanthomonas citri

Xylella fastidiosa

Yersinia pestis Antiqua

Zymomonas mobilis ZM4

Supplemental table 2. ``The top ten genes by number of appearances in triplets with

*∆U* >= 0.3 using balanced profiles and their functions.

| gene name | function | essential gene in *E. coli*? | GI |
| --- | --- | --- | --- |
| ibpA | a small heat shock protein that binds to aggregated proteins and inclusion bodies formed during heterologous protein expression | No | 16131555 |
| ycgN | conserved protein, function unknown | No | 145698245 |
| nadK | an allosteric kinase, with activity tightly coupled to the NADPH/NADP^+^ and NADH/NAD^+^ ratios present in the cell | Yes | 16130534 |
| hemA | glutamyl-tRNA reductase, catalyzes the first step of porphyrin biosynthesis | Yes | 16129173 |
| ptsN | a protein homologous to Enzyme IIA^fru^ of the phosphoenolpyruvate (PEP)-dependent carbohydrate phosphotransferase system (PTS) | No | 16131094 |
| ribE | lumazine synthase, an enzyme that catalyzes the penultimate step in the riboflavin biosynthesis pathway | Yes | 78044703 |
| yqgF | a conserved protein similar to nucleases and Holliday junction resolvase | Yes | 16130850 |
| CHY_0211 | Ppx/GppA phosphatase, similar to gpp in *E. coli*. | No | 78043933 |
| tilS | a tRNA^Ile^-lysidine synthetase, the enzyme responsible for modifying the wobble base of the CAU anticodon of tRNA^Ile^ | Yes | 53804114 |
| APH_0213 | putative phosphoribosylformylglycinamidine synthase II, similar to purL in E. coli. | No | 88606986 |

Supplemental figure 1. Percentage of different logic relationships among the eight types across the whole spectrum of ∆U.

A


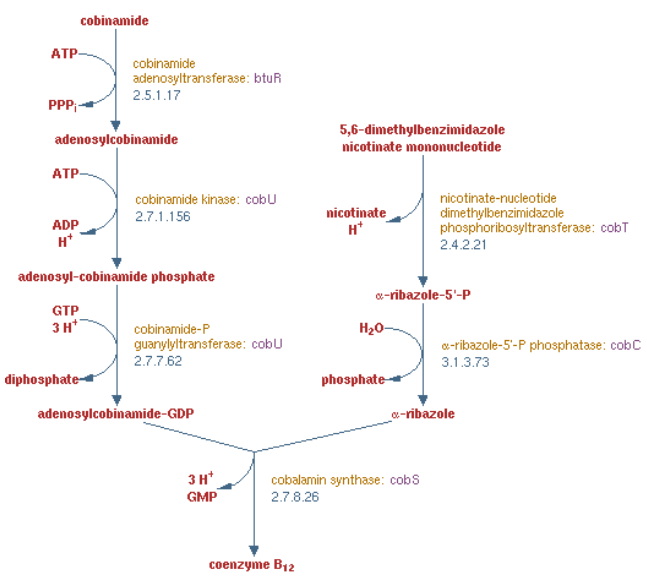
 B


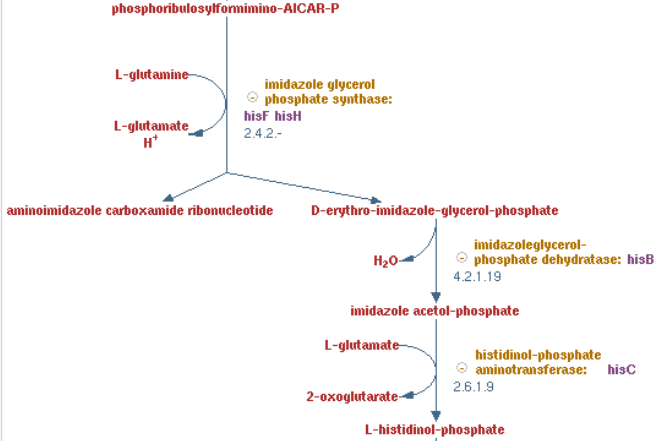


C


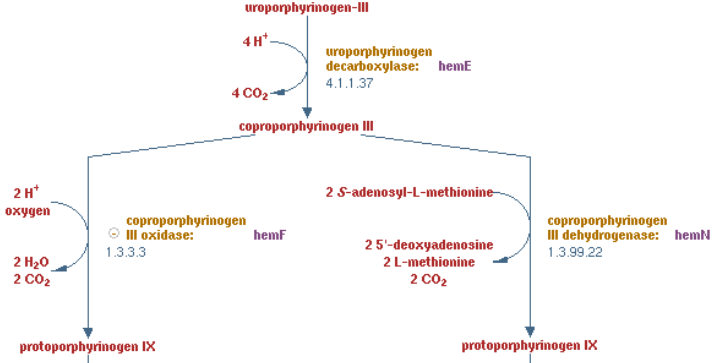


Supplemental figure 2. Some examples of logic triplets in *E. coli* ; **A**: c*obS is present iff cobU and cobC are both present* in the pathway of adenosylcobalamin salvage from cobinamide I. **B**:*hisB is present iff either hisF or hisH is present* in histidine biosynthesis pathway; **C**: *hemE is present iff either hemF or hemN is present* in the superpathway of heme biosynthesis from uroporphyrinogen-III.

Supplemental figure 3. The log number of triplets where a gene is in output role *c* vs. input role *a* or *b*, the base of log is 10. Each dot represents a gene in *E. coli* with red for essential genes and green for non-essential genes; a dot (1.0, 2.0) means the gene appears in 110 triplets where it is at output role in 10 triplets and at input role in 100 triplets. Genes are separated into three groups according to their roles in the triplets: 1) those on *y*-axis, they can only be at input role; 2) those on *x*-axis, they are only at output role; and 3) those in the quadrant where *x* and *y* > 0, they can be at any role. The grouping may reveal the general role of a gene in gene association network; for example, more essential genes are in input role and more non-essentials in output role. This figure demonstrates that some essential genes have only small number of associations with other genes. And as suggested by the enrichment in the strip 2.5 <= y <= 4.0, the chance of a gene being at the output role in a cellular network varies much more than its chance at the input role.

**Gene triplets and gene essentiality**

Logic relationships might infer the importance and position of a gene in the whole gene association network; therefore it is interesting to see whether there is any difference on that regard between the 302 essential genes and 3163 non-essential genes in *E. coli* . With a phylogenetic matrix using *E. coli* as a reference genome, we obtained logic relationships with *∆U*>0.3 involving 139 essential genes and 738 non-essential genes. On average, the essential genes appeared in 1931 triplets per gene vs. 1390 for non-essential genes, but the difference was not significant by Wilcoxon Rank Sum Test. Furthermore, it is evident from Supplemental Figure 3 that some essential genes are involved in very small number of triplets, which reveals that they are at a unique, non-replaceable position although they are not well connected in the association network.

Based on the position in a triplet, genes in Supplemental figure 3 are separated into the following three groups. Chi Square Test between the essentiality of genes and their grouping gave *p* = 4.1 x 10^-5^, a strong indication that the essentiality and the grouping were not independent from each other. But no significant difference was found on the enriched GO terms among the three categories.

1) Genes on *y*-axis, they can only be at *a* or *b* position. This group accounted for 35.1% of non-essential genes but 54.0% of essential genes; it confirms that other genes depend more on the essential genes instead of the other way around. In the cellular network, those genes are possibly located in the initial stages of pathways. For example, in all of the 3584 triplets gene lepB is involved in, it is never in position *c*; it codes for a signal peptidase which cleaves the signal peptides from secretory proteins; since nearly half of proteins are secreted, it explains the large number of dependences. Other similar examples include tRNA synthetase (asnS, argS, metG), initiator proteins for the assembly of the 30S subunit of the ribosome (rpsD), chaperone protein (groS), etc.

2) Genes on *x*-axis, they are only at *c* position. This group accounts for 45.3% of non-essential genes but only 27.3% of essential genes, again indicating that essential genes are less likely to be in the *c* position than other genes. These genes possibly function at the end of the network or pathways. Genes at the top of this group mostly have functions involving ribosome, chromosome, and translocation of proteins through membrane.

3) Genes in the quadrant, the space where *x*, *y* > 0; they can be at any of the three positions. Those genes probably locate in the middle steps of the network, but surprisingly it only covers about 20% of genes in both the essential and non-essential sets. Examples are UDP-2,3-diacylglucosamine hydrolase catalyzing the fourth step in lipid A synthesis (lpxH), N-succinyl-L-diaminopimelate desuccinylase required for the seventh step in lysine biosynthesis (dapE), exonuclease (rdgC), diadenosine tetraphosphatase (apaH), Glyoxalase II (gloB) for the second step in the conversion of methylglyoxal to D-lactate , etc. Interestingly, majority of the genes in this group clustered between *y* of [2.5, 4.0] whereas the *x* values spread almost evenly within [0.25, 3.5]; it suggests that the upstream dependencies of a gene in this group is much more variable than its downstream relationships in a cellular network.

In addition, if genes in at least one triplet with *∆U* > 0.3 are put in *selected* category, and the remaining genes are in *non-selected* category, essential genes are highly significant (p-value =2.1 x 10^-16^) to be in the selected category by applying Fisher’s Exact Test to the following contingency table (This analysis is contributed by a reviewer).

|  | selection | nonselection | Sum | percentage |
| --- | --- | --- | --- | --- |
| essential gene | 139 | 163 | 302 | 8.70% |
| non-essential gene | 738 | 2,425 | 3,163 | 91.30% |
| Sum | 877 | 2,588 | 3,465 |  |
| percentage | 25.30% | 74.70% |  |  |

Conversely, logic triplets can provide clues on the essentiality of genes and gene combinations, which are critical information for the construction of cellular network. For example, for relationship “*c* is present iff *a* and *b* are both present”, if *c* is essential, *a* and *b* must be essential too; for relationship “*c* is present iff *a* or *b* is present”, if *c* is essential, *ab* combination must be essential as well although individually they may not.

**Enriched genes and GO terms**

The top ten genes with most triplets are listed in Supplemental table 2. Not surprisingly, they either serve in some fundamental steps or catalyze very common reactions across pathways. Only half are essential genes, proving that essential genes are not necessary involved in more associations with other genes even if they are vital to cell function.

Overall, the top GO terms are concentrated in cellular biosynthetic process; cellular macromolecule metabolic process, especially those involving nitrogen compound and nucleic acid; gene expression; carboxylic acid biosynthetic process; translation; transcription; cofactor metabolic process, etc. However, their associations with *∆U* vary. Triplets in cellular biosynthetic process mostly have low *∆U*, and those in cellular nitrogen compound metabolic process, particularly nucleobase, nucleoside, nucleotide and nucleic acid metabolic process have high *∆U*. Due to the underlying condition to achieve high significance of triplets explained in the Discussion section, GO terms prominent in low *∆U* may have denser and more variable local network across species than those prominent in high *∆U*.

**The** **bi-directionality of the *iff* condition in a logic triplet**

Each of the eight logic relationships in Table 1 can be represented by four *abc* combinations, and each combination is bi-directional between *ab* and *c*, meaning that *ab* determines *c*, and *c* can determine *ab* as well. Taking the type 3 relationship as an example, it corresponds to the combination 000, 011, 101, 111. Following is the proof of the bi-directionality in the four columns. *iff* consists of two parts: *if*, and *only if*.

| 1. if | *a* | or | *b*, | then | *c*. | contrapositive: | if !*c*, | then | !*a* | and | !*b* |
| --- | --- | --- | --- | --- | --- | --- | --- | --- | --- | --- | --- |
|  | **1** |  | **0** |  | **1** |  | **0** |  | **0** |  | **0** |
|  | **0** |  | **1** |  | **1** |  |  |  |  |  |  |
|  | **1** |  | **1** |  | **1** |  |  |  |  |  |  |
|  | | | | | | | | | | | |
| 2. "only if *a* or *b*, then *c*" is equivalent to "if *c*, then *a* or *b*". | | | | | | | | | | | |
| if | *c*, | then | *a* | or | *b*. | contrapositive: | if !*a* | and | !*b*, | then | !*c* |
|  | **1** |  | **1** |  | **0** |  | **0** |  | **0** |  | **0** |
|  | **1** |  | **0** |  | **1** |  |  |  |  |  |  |
|  | **1** |  | **1** |  | **1** |  |  |  |  |  |  |

In the forward direction, *c* is determined once *ab* is given. In the reverse direction, *ab* is determined to be 00 if *c* is 0, and *ab* is determined to be {01,10,11} if *c* is 1.

In comparison, in a one-directional “*c* if *a* or *b*” logic type, *ab* is NOT determined if *c* is 1; for example, *ab* may be {11}, or {11,01}, etc.

The bi-directionality of other logic relationships can be proven similarly.

**Statistical significance of *∆U***

Phylogenetic profiling for functional linkage analysis in a whole genome often needs a threshold value for the score signaling the confidence level of the linkage, and the threshold is usually determined by the evaluation of its probability to appear from profiles of unrelated genes. To construct such hypothetical profiles, randomly shuffling of existing profiles is usually applied . However randomization has two assumptions that are not true in a real profile matrix: i) each gene has an equal chance to be present or absent in any genome; ii) genomes in the matrix are totally independent from each other. As a result, such method tends to over-estimate the statistic significance of a score .

The shuffling can be more accurate if it can avoid the two assumptions by keeping the frequency of 0 and 1 in each column and in each large clade at each row. Here we use random set of genes from the existing matrix to compute the statistic significance of different thresholds. Subsequently we computed the *∆U* of nine billion random triplets. *∆U* ≥ 0.3, the threshold value used in Bowers et al., had a p-value of 0.00024. The p-value was good enough that we decided to use the same threshold for our study. The reason that such low p-value only corresponds to 30% putative triplets in Figure 1A could be due to firstly the errors and incompleteness in GO annotation, and secondly the unrelated genes with the very similar profiles. It should also be noted that thresholds are generally specific to the dataset (i.e., phylogenetic matrix) and their p-values should be re-investigated whenever the dataset changes.

**Reference**
